# Supplementary material for: The phylogeography and incidence of multi-drug resistant typhoid fever in sub-Saharan Africa
Source: Nat Commun. 2018 Nov 30;9:5094. doi: 10.1038/s41467-018-07370-z (PMC6269545; doi:10.1038/s41467-018-07370-z)
Supplement: Supplementary file 1 — Supplementary Information [file 41467_2018_7370_MOESM1_ESM.pdf]

Park et al., The phylogeography and incidence of multi-drug resistant typhoid fever in sub-Saharan Africa.

Supplementary Information

- **Supplementary Table 1.**
  Distribution of major MDR *S.* Typhi genotypes circulating in sub-Saharan Africa

- **Supplementary Table 2.**
  All *Salmonella* Typhi organisms analysed in this manuscript

- **Supplementary Figure 1.**
  Root-to-tip regression for the subclade 3.1.1 maximum likelihood tree

- **Supplementary Figure 2.**
  Date-randomization test for subclade 3.1.1 using in BEAST2

- **Supplementary Figure 3.**
  Estimates of the time to the most recent common ancestor of the 3.1.1 lineage using BEAST2 and LSD

Supplementary Table 1. Distribution of major MDR *S.* Typhi genotypes circulating in sub-Saharan Africa

Country (total no. of *S.* Typhi)	Genotypes	No. of *S.* Typhi	% of total no. of *S.* Typhi per country
Burkina Faso (14)	2.2	2	14
	2.3.2	2	14
	3.1.1	8	57
	4.1.1	2	14
Ethiopia (2)	1.2	1	50
	2.2.2	1	50
Gambia (11)	2.3.2	11	100
Ghana (101)	0.0.1	1	1
	0.0.3	2	2
	2.3.2	8	8
	3.1.1	89	88
	4.1	1	1
Guinea-Bissau (3)	2.3.1	1	33
	2.3.2	2	67
Kenya (59)	4.3.1	59	100
Madagascar (8)	2.2	3	38
	2.5	4	50
	4.1	1	13
Senegal (8)	2.3.2	6	75
	3.1	1	13
	4.1	1	13
South Africa (2)	3.3.1	2	100
Tanzania (11)	2.2.2	1	9
	4.3.1	10	91
Uganda (30)	4.3.1	30	100

Genotypes (number of isolates)	Region	Country	Genotype per country	MDR[1] per country
4.3.1 (99)	East Africa	Kenya (n=59)	60% (59/99)	85% (50/59)
		Uganda (n=30)	30% (30/99)	23% (7/30)
		Tanzania (n=10)	10% (10/99)	40% (4/10)
3.1.1 (97)	West Africa	Ghana (n=89)	92% (89/97)	76% (68/89)
		Burkina Faso (n=8)	8% (8/97)	0% (0/8)
2.3.2 (29)	West Africa	Gambia (n=11)	38% (11/29)	0%
		Ghana (n=8)	28% (8/29)	0%
		Senegal (n=6)	21% (6/29)	0%
		Burkina Faso (n=2)	7% (2/29)	0%
		Guinea Bissau (n=2)	7% (2/29)	0%

Genotype	Number of isolates	MDR (%) per genotype
4.3.1 (H58)	99 (40%)	62% (61/99)
3.1.1	97 (39%)	70% (68/97)
2.3.2	29 (12%)	0%
Other minor genotypes[2]	24 (10%)	0%
Total	249 (100%)	52% (129/249)

[1] MDR definition used for the analysis: presence of resistant genes for at least one agent in all three antimicrobial categories listed below (detected in this study)

- Ampicillin/amoxicillin: beta-lactamase (*OXA-1, TEM-95/-93*)
- Chloramphenicol: (*catA1*)
- Trimethoprim-sulfamethoxazole: sulfonamide (*sul1, sul2*) and trimethoprim (*dfrA7, dfrA14, dfrA15*)

[2] Other minor genotypes of *S.* Typhi organisms (genotype distribution per country):

- 2.2 Total 7 *S.* Typhi: 1/2 (50%) Ethiopia, 3/8 (38%) Madagascar, 2/14 (14%) Burkina Faso, 1/11 (9%) Tanzania
- 4.1.1 Total 5 *S.* Typhi: 2/14 (14%) Burkina Faso, 1/8 (13%) Senegal, 1/8 (13%) Madagascar, 1/101 (1%) Ghana
- 2.5 Total 4 *S.* Typhi: 4/8 (50%) Madagascar
- 3.3.1 Total 2 *S.* Typhi: 2/2 (100%) South Africa
- 0.0.3 Total 2 *S.* Typhi: 2/101 (2%) Ghana
- 1.2 Total 1 *S.* Typhi: 1/2 (50%) Ethiopia
- 2.3.1 Total 1 *S.* Typhi: 1/3 (33%) Guinea-Bissau
- 3.1 Total 1 *S.* Typhi: 1/8 (13%) Senegal
- 0.0.1 Total 1 *S.* Typhi: 1/101 (1%) Ghana

Supplementary Table 2. All *Salmonella* Typhi organisms analysed in this manuscript

	Burkina Faso	Ethiopia (Butajira)	Ghana	Guinea-Bissau (Bandim)	Kenya (Kibera)	Madagascar	Senegal (Pikine)	South Africa	Tanzania	Uganda	Gambia
Isolates analyzed for this study[1] (249 *S.* Typhi)	14	2	101	3	59	8	8	2	11	30	11
	11 (Polesgo)<br>3 (NiokoII)	2	30 (TSAP)<br>11 (TSAP; outside catchment area)<br>22 (TSAP; >15yrs)<br>6 (“IsolateAgogo”)<br>32 (unidentified)	3	59	6 (Imerintsiatosika)<br>2 (Isotry)	8	2	6 MU[4]<br>4 MR[5]<br>1 MO[6]		
TSAP[2] (135 *S.* Typhi)	18	3	30	3	54	9	7	2	9	n.a.	n.a.
	13 (Polesgo)<br>5 (NiokoII)	3	30 (AAN)[3]	3	54	6 (Imerintsiatosika)<br>3 (Isotry)	7	2	6 (18) MU[4]<br>3 (6) MR[5]	n.a.	n.a.
Other_Ghana[7]	n.a.	n.a.	6 (“IsolateAgogo”)<br>32 (unidentified)	n.a.	n.a.	n.a.	n.a.	n.a.	n.a.	n.a.	n.a.
Other_Uganda	n.a.	n.a.	n.a.	n.a.	n.a.	n.a.	n.a.	n.a.	n.a.	30	n.a.
Other_Gambia	n.a.	n.a.	n.a.	n.a.	n.a.	n.a.	n.a.	n.a.	n.a.	n.a.	11

[1] Total 249 *S.* Typhi organisms were analyzed for this study: 208 *S.* Typhi isolates from TSAP and other Ghana projects; 30 *S.* Typhi from Uganda; and 11 *S.* Typhi from Gambia.

[2] TSAP recruited 13,558 patients from 13 sites in 10 countries meeting the study inclusion criteria, of which 127 patients were excluded due to incomplete dataset. This resulted in 13,431 patients and 135 *S.* Typhi found in 9 countries for analysis (Marks et al. Lancet Global Health 2017). No *S.* Typhi was found from Sudan. The differences in the number of *S.* Typhi in TSAP publication (total 135 *S.* Typhi in Marks et al) and the isolates analyzed for this manuscript (total 249 *S.* Typhi, including 208 *S.* Typhi from TSAP and other Ghana projects, 30 and 11 *S.* Typhi organisms from Uganda and Gambia, respectively) are due to several factors listed below:

- Kenya, Senegal, and Tanzania: *S.* Typhi cases, which did not meet the catchment area or fever recruitment criteria, were excluded in the final analysis in the TSAP publication but included in this bacterial genomic analysis.
- Burkina Faso, Ethiopia, and Madagascar: *S.* Typhi isolates analyzed for this study are based on the whole genome sequencing result and quality control screening.
- Ghana: 30 *S.* Typhi isolates from under 15 years of age were presented in TSAP publication. Additionally, 11 isolates from TSAP but not meeting the catchment area criteria, 22 isolates from TSAP but above 15 years of age, 6 isolates from “IsolateAgogo” dataset, and 32 isolates from non-TSAP projects in Ghana (“FISA”, “TYSA”, and “IsolateAgogo”) have been added to this analysis.

[3] AAN: Asante Akim North

[4] MU: Moshi Urban

[5] MR: Moshi Rural

[6] MO: Moshi Other (This 1 case was out of the TSAP catchment area; 21 year old patient enrolled with *S.* Typhi infection.)

[7] Other_Ghana: *S.* Typhi isolates from non-TSAP projects in Ghana (“Febrile Illnesses Surveillance in Africa (FISA)”, “Typhoid Surveillance in Africa (TYSA)”, and “IsolateAgogo” datasets)

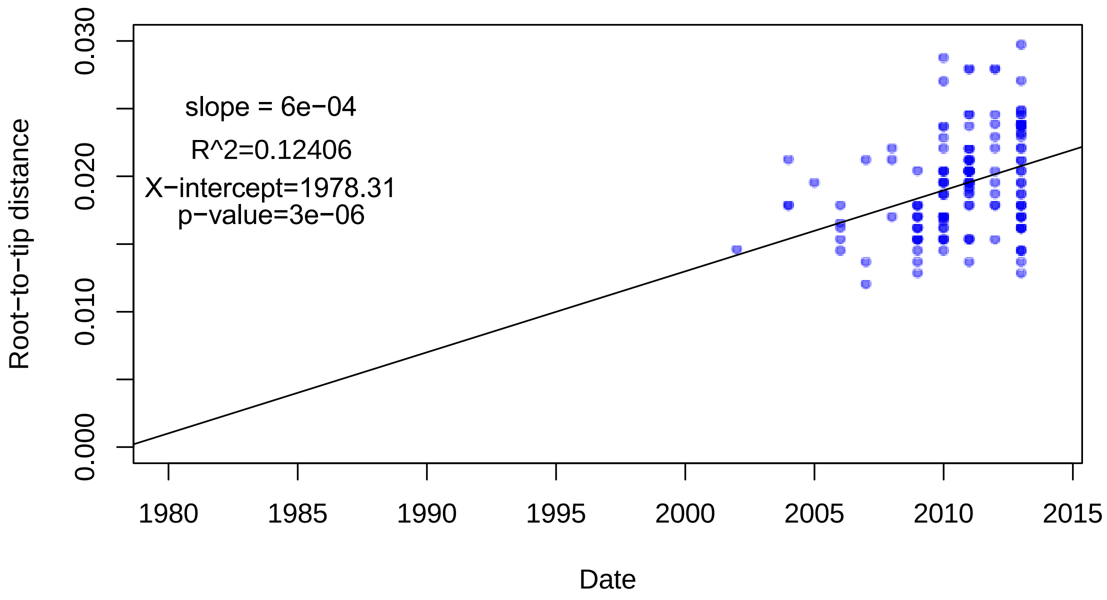

Supplementary Figure 1. Root-to-tip regression for the subclade 3.1.1 maximum likelihood tree

Each point on the plot corresponds to a measurement from the root to each tip in the tree. The solid line is the regression line fitted using the ordinary least squares method. The slope of the line is a crude estimate of the evolutionary rate, the x-intercept corresponds to the time to the most recent common ancestor, and the R^2 value measures the degree of clocklike behavior. Note that the *y*-axis is the root-to-tip distance for the SNP alignment, such that the slope corresponds to the SNP rate estimate.

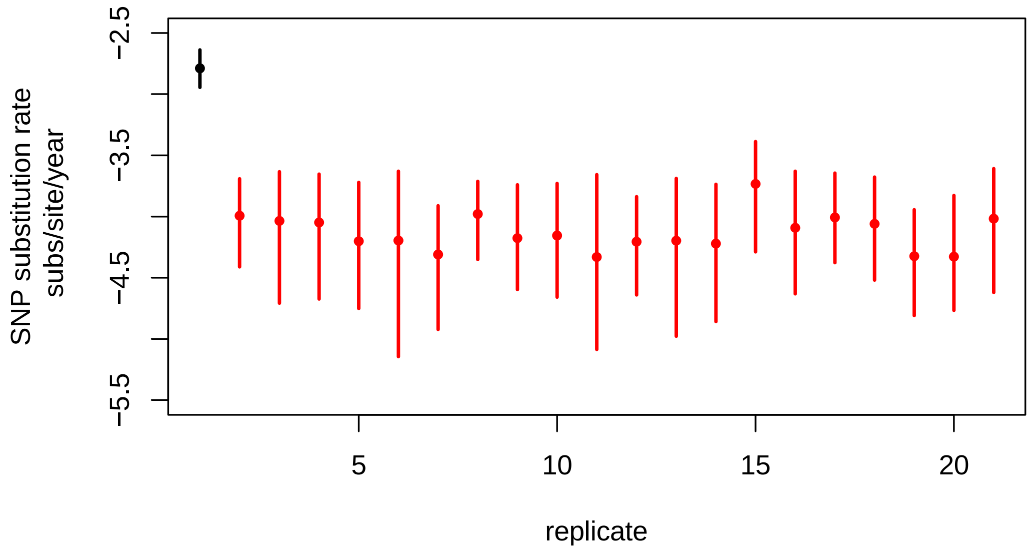

Supplementary Figure 2. **Date-randomization test for subclade 3.1.1 using in BEAST2**

The *y*-axis shows the SNP evolutionary rate in $\log_{10}$ scale, while the *x*-axis shows the different replicates of the date-randomization test. Points are mean estimates and the lines are the Bayesian credible intervals around these values. The black symbol is for the data with real sampling times, while those in red are for the date-randomizations. The data are considered to display strong temporal structure if the estimate with the correct sampling times does not overlap with those from the randomizations.

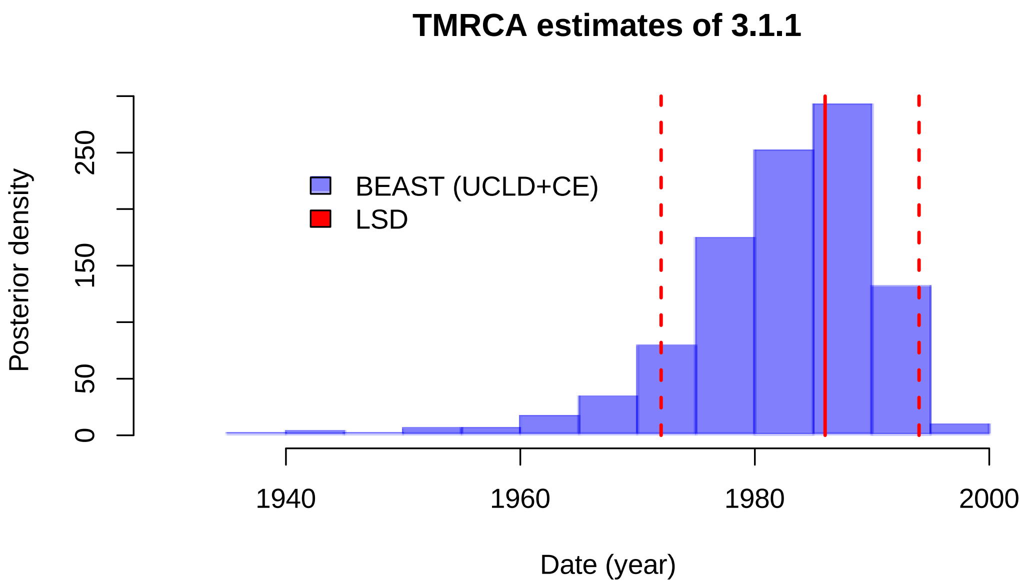

Supplementary Figure 3. Estimates of the time to the most recent common ancestor of the 3.1.1 lineage using BEAST2 and LSD

Blue histogram corresponds to the posterior distribution in BEAST2 using the uncorrelated lognormal clock model and the exponential growth coalescent tree prior. The red lines represent the estimate from LSD (Least-squares dating), where the solid line is the mean and the dashed lines are the 95% confidence interval.
