# Supplementary material for: The phylogeography and incidence of multi-drug resistant typhoid fever in sub-Saharan Africa
Source: Nat Commun. 2018 Nov 30;9:5094. doi: 10.1038/s41467-018-07370-z (PMC6269545; doi:10.1038/s41467-018-07370-z)
Supplement: Supplementary file 3 — Reporting Summary [file 41467_2018_7370_MOESM3_ESM.pdf]

nature research

NCOMMS-18-13127B

Corresponding author(s): Florian Marks

Reporting Summary

Nature Research wishes to improve the reproducibility of the work that we publish. This form provides structure for consistency and transparency in reporting. For further information on Nature Research policies, see Authors & Referees and the Editorial Policy Checklist.

Statistical parameters

When statistical analyses are reported, confirm that the following items are present in the relevant location (e.g. figure legend, table legend, main text, or Methods section).

n/a	Confirmed	
☐	☒	The exact sample size (n) for each experimental group/condition, given as a discrete number and unit of measurement
☐	☒	An indication of whether measurements were taken from distinct samples or whether the same sample was measured repeatedly
☐	☒	The statistical test(s) used AND whether they are one- or two-sided<br>*Only common tests should be described solely by name; describe more complex techniques in the Methods section.*
☐	☒	A description of all covariates tested
☐	☒	A description of any assumptions or corrections, such as tests of normality and adjustment for multiple comparisons
☐	☒	A full description of the statistics including central tendency (e.g. means) or other basic estimates (e.g. regression coefficient) AND variation (e.g. standard deviation) or associated estimates of uncertainty (e.g. confidence intervals)
☐	☒	For null hypothesis testing, the test statistic (e.g. F, t, r) with confidence intervals, effect sizes, degrees of freedom and P value noted<br>*Give P values as exact values whenever suitable.*
☐	☒	For Bayesian analysis, information on the choice of priors and Markov chain Monte Carlo settings
☐	☒	For hierarchical and complex designs, identification of the appropriate level for tests and full reporting of outcomes
☐	☒	Estimates of effect sizes (e.g. Cohen's d, Pearson's r), indicating how they were calculated
☐	☒	Clearly defined error bars<br>*State explicitly what error bars represent (e.g. SD, SE, CI)*

Our web collection on statistics for biologists may be useful.

Software and code

Policy information about availability of computer code

Data collection	No software was used for data collection.
Data analysis	SMALT v0.7.4; SAMtools; Gubbins; RAxML v8.2.8; BEAST2 v2.4.7; TempEst; LSD v0.3; ARIBA; VelvetOptimiser v2.2.5; Velvet v1.2,Prokka v1.5; SRST2; SPADES v3.11; Bandage

For manuscripts utilizing custom algorithms or software that are central to the research but not yet described in published literature, software must be made available to editors/reviewers upon request. We strongly encourage code deposition in a community repository (e.g. GitHub). See the Nature Research guidelines for submitting code & software for further information.

Data

Policy information about availability of data

All manuscripts must include a data availability statement. This statement should provide the following information, where applicable:

- Accession codes, unique identifiers, or web links for publicly available datasets
- A list of figures that have associated raw data
- A description of any restrictions on data availability

Raw sequence data are available in the European Nucleotide Archive (projects ERP009684, ERP010763, ERP013866). The Microreact interactive phylogeny of the analysed isolates is available at: https://microreact.org/project/HJWBihsvz. SMALT version 0.7.4 used is available at: http://www.sanger.ac.uk/resources/software/smalt/.

Field-specific reporting

Please select the best fit for your research. If you are not sure, read the appropriate sections before making your selection.

☒ Life sciences ☐ Behavioural & social sciences ☐ Ecological, evolutionary & environmental sciences

For a reference copy of the document with all sections, see nature.com/authors/policies/ReportingSummary-flat.pdf

Life sciences study design

All studies must disclose on these points even when the disclosure is negative.

Sample size	A population-based surveillance of febrile patients was conducted in pre-defined catchment areas covered by the selected sentinel healthcare facilities. Sample size corresponded to the population size of the pre-defined study catchment areas.
Data exclusions	No data were excluded from the analyses.
Replication	*Describe the measures taken to verify the reproducibility of the experimental findings. If all attempts at replication were successful, confirm this OR if there are any findings that were not replicated or cannot be reproduced, note this and describe why.*
Randomization	Randomization was not relevant to our prospective population-based observational study
Blinding	Group allocation and blinding were not applied in our data collection and analyses.

Reporting for specific materials, systems and methods

Materials & experimental systems

n/a	Involved in the study
☒	☐ Unique biological materials
☒	☐ Antibodies
☒	☐ Eukaryotic cell lines
☒	☐ Palaeontology
☒	☐ Animals and other organisms
☐	☒ Human research participants

Methods

n/a	Involved in the study
☒	☐ ChIP-seq
☒	☐ Flow cytometry
☒	☐ MRI-based neuroimaging

Human research participants

Policy information about studies involving human research participants

Population characteristics	All febrile patients regardless of ages (except in Ghana where only children aged <15 years were enrolled) or gender or any past and current diagnosis and treatments were eligible for recruitment.
Recruitment	All eligible patients meeting the study inclusion criteria (fever measured with a tympanic or axillary temperature equal to or above 38.0c or 37.5c, respectively, living in the defined study areas; for inpatients, reported fever within the period of 72 hours prior to admission was added to the inclusion criteria) were approached for informed consent. Recruitment into the surveillance study took place after the informed consent process.
